# Comparison of a second-generation trabecular bypass (iStent inject) to ab interno trabeculectomy (Trabectome) by exact matching

**DOI:** 10.1007/s00417-020-04933-z

**Published:** 2020-09-22

**Authors:** Yousef Al Yousef, Alicja Strzalkowska, Jost Hillenkamp, André Rosentreter, Nils A. Loewen

**Affiliations:** 1 Department of Ophthalmology, University of Würzburg, Josef-Schneider-Straße, 11 97080 Würzburg, Germany; 2 Department of Ophthalmology, University Witten/Herdecke, Wuppertal, Germany

**Keywords:** Glaucoma surgery, iStent, Trabecular bypass stent, Trabectome, Ab interno trabeculectomy, Exact matching

## Abstract

**Purpose:**

To achieve a highly balanced comparison of trabecular bypass stenting (IS2, iStent inject) with ab interno trabeculectomy (T, Trabectome) by exact matching.

**Methods:**

Fifty-three IS2 eyes were matched to 3446 T eyes. Patients were matched using exact matching by baseline intraocular pressure (IOP), the number of glaucoma medications, and glaucoma type, and using nearest neighbor matching by age. Individuals without a close match were excluded. All surgeries were combined with phacoemulsification.

**Results:**

A total of 78 eyes (39 in each group) could be matched as exact pairs with a baseline IOP of 18.3 ± 5.1 mmHg and glaucoma medications of 2.7 ± 1.2 in each. IOP in IS2 was reduced to 14.6 ± 4.2 mmHg at 3 months and in T to a minimum of 13.1 ± 3.2 mmHg at 1 month. In IS2, IOP began to rise again at 6 months, eventually exceeding baseline. At 24 months, IOP in IS2 was 18.8 ± 9.0 mmHg and in T 14.2 ± 3.5 mmHg. IS2 had a higher average IOP than T at all postoperative visits (*p* < 0.05 at 1, 12, 18 months). Glaucoma medications decreased to 2.0 ± 1.5 in IS2 and to 1.5 ± 1.4 in T.

**Conclusion:**

T resulted in a larger and sustained IOP reduction compared with IS2 where a rebound occurred after 6 months to slightly above preoperative values.

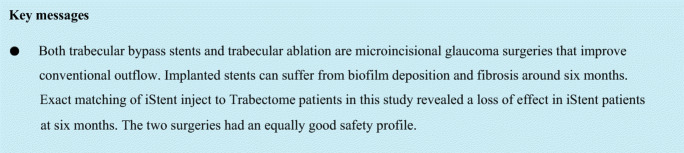

## Introduction

Following reports of fibrosis [1] and biofilm deposition [2] after trabecular bypass device implantation, we recently used exact matching to compare a first-generation trabecular bypass stent (IS1, iStent, Glaukos Corp., San Clemente, CA) to trabecular ablation (T, Trabectome, MicroSurgical Technology, Redmond, WA) [3]. Many surgeons now use microincisional glaucoma surgeries (MIGS) first before considering more extensive, traditional procedures (trabeculectomy, tube shunt) because they have a lower complication rate [4] and shorter procedure time, which allows them to be combined with outpatient cataract surgery. In eyes with stents, we found evidence of declining function in the form of increasing IOP and glaucoma medications above preoperative levels while IOP and medications reduced in matched eyes that had undergone trabecular meshwork (TM) ablation [3]. In the present study, we examined whether a smaller, second-generation trabecular bypass stent (IS2, iStent inject, Glaukos Corp., San Clemente, CA) would behave differently. Both the IS1 and the IS2 are made of heparin-coated titanium, but two IS2s are implanted in one session instead of just one IS1. We compared IS2 with T using exact matching, an advanced statistics method developed for a highly balanced comparison [3, 5, 6]. We hypothesized that this method would again reveal significant effect differences between IS2 and T that can be missed in studies that use group averages [7, 8] and may be caused by chronic responses at that the implant site [9–12].

## Methods

### Study design

The study was approved by the local ethics committee of the University of Würzburg (protocol #20191016 01). Because of its retrospective nature, informed consent was waived. The study was in accordance with the 1964 Helsinki declaration and its later amendments or comparable ethical standards. This study included all patients who underwent either IS2 or T in our clinic and associated satellites between January 2008 and March 2018. The indication for surgery was a stable IOP with a desire to reduce glaucoma medications at the time of cataract surgery or an above-target IOP as determined by a glaucoma specialist, while on maximally tolerated topical treatment. To increase the chances of an exact match to the new IS2, of which fewer data exist, data from the Trabectome Study Group database [13, 14] was used to bring the number of T available for an exact match to 3446. We excluded patients younger than 20 years of age, with neovascular or uveitic glaucoma, uncontrolled uveitis, or prior ocular surgery. At baseline, we assessed patient history and obtained demographic data, type, and stage of glaucoma; best-corrected visual acuity (BCVA); intraocular pressure (IOP); and the number of glaucoma medications. At each follow-up visit, we recorded BCVA, IOP, and the number of glaucoma medications. The primary outcome was a ≥ 20% reduction of IOP compared with baseline or an IOP of less than 21 mmHg to better reflect the most common real-world use pattern of the IS2 as an add-on surgery at the time of cataract surgery rather than a principal glaucoma surgery. We combined both IS2 and T with phacoemulsification and intraocular lens implantation in all cases. The treating specialists could decide what glaucoma drops to use or whether another glaucoma surgery was needed.

### Statistics

Data were described as frequency, percentage, mean ± SD, median, and range. Continuous and categorical variables were compared with the Mann-Whitney *U* test and chi-squared test. Using exact matching, both groups were matched using preoperative IOP, glaucoma medications, and type of glaucoma, and using Nearest Neighbor Matching for age [15]. Each unit in group 1 (IS2) was matched using exact matching to all possible control units in group 2 (T), whereas nearest neighbor matching selected the best matches based on the distance to the value in group 1. *p* values of less than 0.05 were considered statistically significant. Mean ± SD was used to express continuous variables. Statistical analyses were performed using R [16].

### Surgical technique

IS2 and T were combined with phacoemulsification and lens implantation in all cases. IS2 implantation was done after cataract surgery, while T was done first and followed by cataract surgery. IS2 was implanted through a temporal clear corneal incision and with viscoelastic [17]. Schlemm’s canal was identified by allowing blood from the episcleral veins to reflux in relative hypotony. Under direct gonioscopic view, the tip of the inserter was placed against the nasal TM. The stents were inserted by piercing through the TM into Schlemm’s canal before releasing. The inserter was retracted, and the viscoelastic removed [17].

T was performed as described before [18]. Briefly, a 1.6-mm uniplanar, temporal clear corneal incision was created. Under direct gonioscopic visualization, the tip of the handpiece was inserted into Schlemm’s canal, and the TM was ablated counterclockwise, followed by clockwise ablation with a total length of around 120° [19–21]. Ablation was started with the power set to 0.8 mW and increased as necessary. The handpiece was withdrawn from the anterior chamber. T was done before cataract surgery to provide the highest corneal transparency for angle surgery and because this incision is smaller than the one needed for cataract surgery. After T, a viscoelastic device was injected to form the anterior chamber before enlarging the clear corneal incision for cataract surgery.

In both IS2 and T, postoperative treatment comprised a topical antibiotic for 1 week and a steroid tapered over 4 weeks. Glaucoma medications were stopped on the day of surgery and restarted as needed.

## Results

**Fig. 1:**
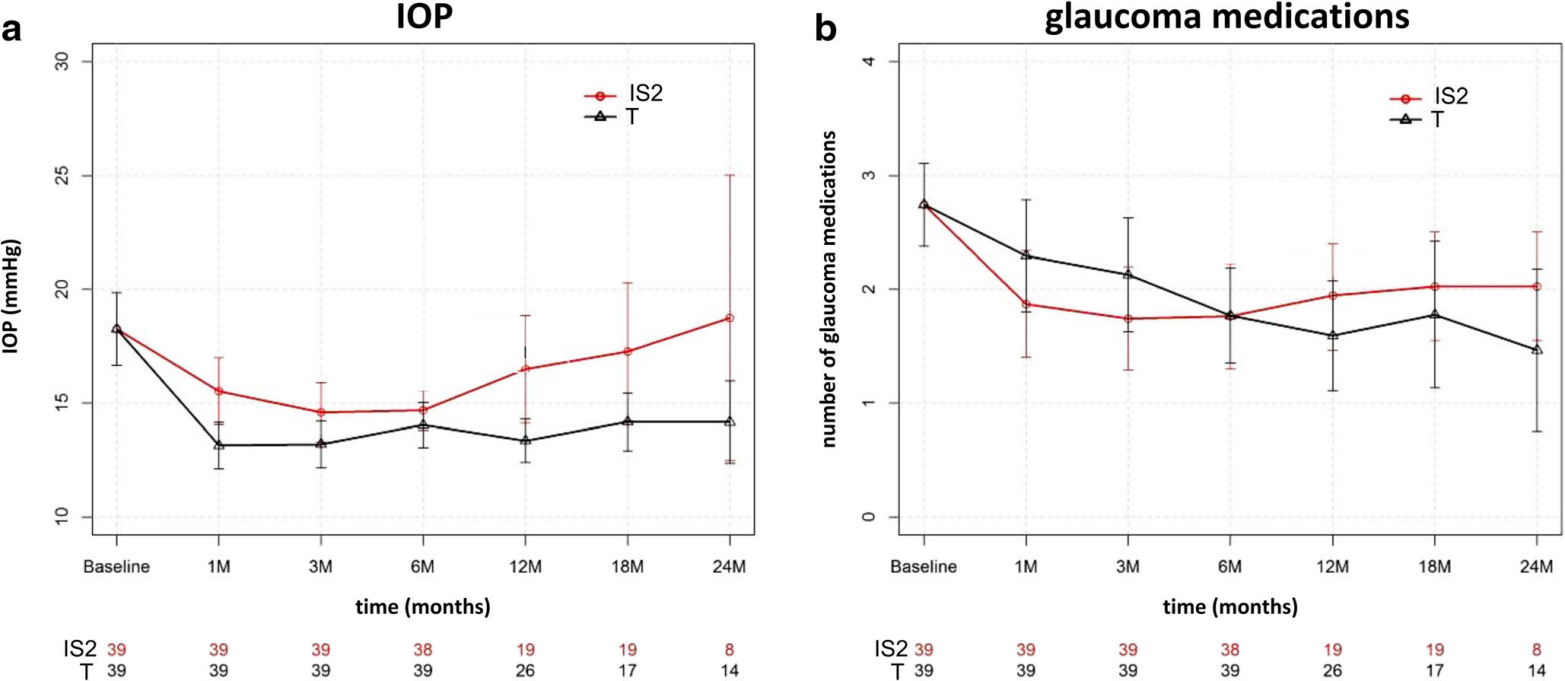
Mean IOP and number of glaucoma medications for IS2 (iStent inject) and T (trabectome). **a** IOP decreased in IS2 through month 3 before starting to rise. In T, IOP decreased through month 1 and remained at this level throughout the study. **b** Glaucoma medications decreased in IS2 before starting to rise again after month 6. In T, medications decreased throughout the study (mean ± SD; subject count at each time point)

Of the 53 IS2 eyes, 39 could be matched to T to create 39 near-identical pairs. Due to exact matching, there was no difference in IOP, the number of IOP-lowering medications, glaucoma type, or VF loss between groups (*p* > 0.05). Table 1 shows the baseline characteristics of each group. IS2 and T had the same preoperative IOP of 18.3 ± 5.1 mmHg. IS2 reached an IOP trough of 14.6 ± 4.2 at 3 months (*p* = 0.04 compared with preoperative). IOP continued to slowly increase in IS2 from 6 months onward, eventually reaching the baseline average (*p* > 0.05, Fig. 1a). T had a minimum IOP of 13.1 ± 3.2 mmHg at 1 month (*p* = 0.01, Fig. 1a) and stayed at that level (*p* < 0.02 all time points compared with preoperative). At 24 months, IOP in IS2 was 18.8 ± 9.0 mmHg, while in T, it was 14.2 ± 3.5 mmHg. T had a lower average IOP than IS2 at all postoperative visits (*p* < 0.05 at 1, 12, 18 months, Table 2).

**Table 1:** Demographics

	T, *n* = 39	IS2, *n* = 39
Age		
Mean ± SD	73 ± 10	72 ± 8
Range	(52, 91)	(57, 84)
Gender		
Female	21 (54%)	22 (56%)
Male	16 (41%)	17 (44%)
NR	2 (5%)	0 (0%)
Diagnosis		
POAG	25 (64%)	25 (64%)
Pseudoexfoliation glaucoma	11 (28%)	11 (28%)
Angle closure glaucoma	1 (3%)	1 (3%)
Normal tension glaucoma	2 (5%)	2 (5%)
Prior surgeries		
ALT	1 (3%)	3 (8%)
SLT	7 (18%)	3 (8%)
Trabeculectomy	0 (0%)	0 (0%)
Combined surgeries		
Trabectome + phaco	39 (100%)	39 (100%)

**Table 2:** Mean IOP and number of medication for Trabectome and iStent groups at each follow-up time point (Welch two-sample *t* test, significance level set at ≤ 0.05)

Time	IOP mean ± SD		*p* value	Rx mean ± SD		*p* value
	T	IS2		T	IS2	
Baseline	18.3 ± 5.1	18.3 ± 5.1		2.7 ± 1.2	2.7 ± 1.2	
1 month	13.1 ± 3.2	15.5 ± 4.7	< 0.01*	2.3 ± 1.6	1.9 ± 1.5	0.89
3 months	13.2 ± 3.3	14.6 ± 4.2	0.05	2.1 ± 1.6	1.7 ± 1.4	0.87
6 months	14.0 ± 3.2	14.7 ± 2.8	0.17	1.8 ± 1.3	1.8 ± 1.5	0.51
12 months	13.3 ± 2.5	16.5 ± 5.2	0.01*	1.6 ± 1.3	1.9 ± 1.5	0.16
18 months	14.2 ± 2.7	17.3 ± 6.8	0.04*	1.8 ± 1.4	2.0 ± 1.5	0.27
24 months	14.2 ± 3.5	18.8 ± 9.0	0.10	1.5 ± 1.4	2.0 ± 1.5	0.11

Glaucoma medications started at a matched value of 2.7 ± 1.2 in IS2 and T. In IS2, they decreased to 1.7 ± 1.4 by month three (*p* = 0.04), increased to a slightly higher average starting at 6 months, and became similar preoperative counts by month 12 (*p* > 0.05). This medication increase did not prevent the IOP from rising. At 24 months, the medication count in IS2 was 2.0 ± 1.5 (*p* > 0.05 compared with preoperative, Fig. 1b). In T, medications decreased from the matched count of 2.7 ± 1.2 at baseline, became significantly lower at 6 months (1.6 ± 1.3, *p* = 0.03), and declined to 1.5 ± 1.4 at 24 months (*p* = 0.04, Fig. 1b).

**Fig. 2:**
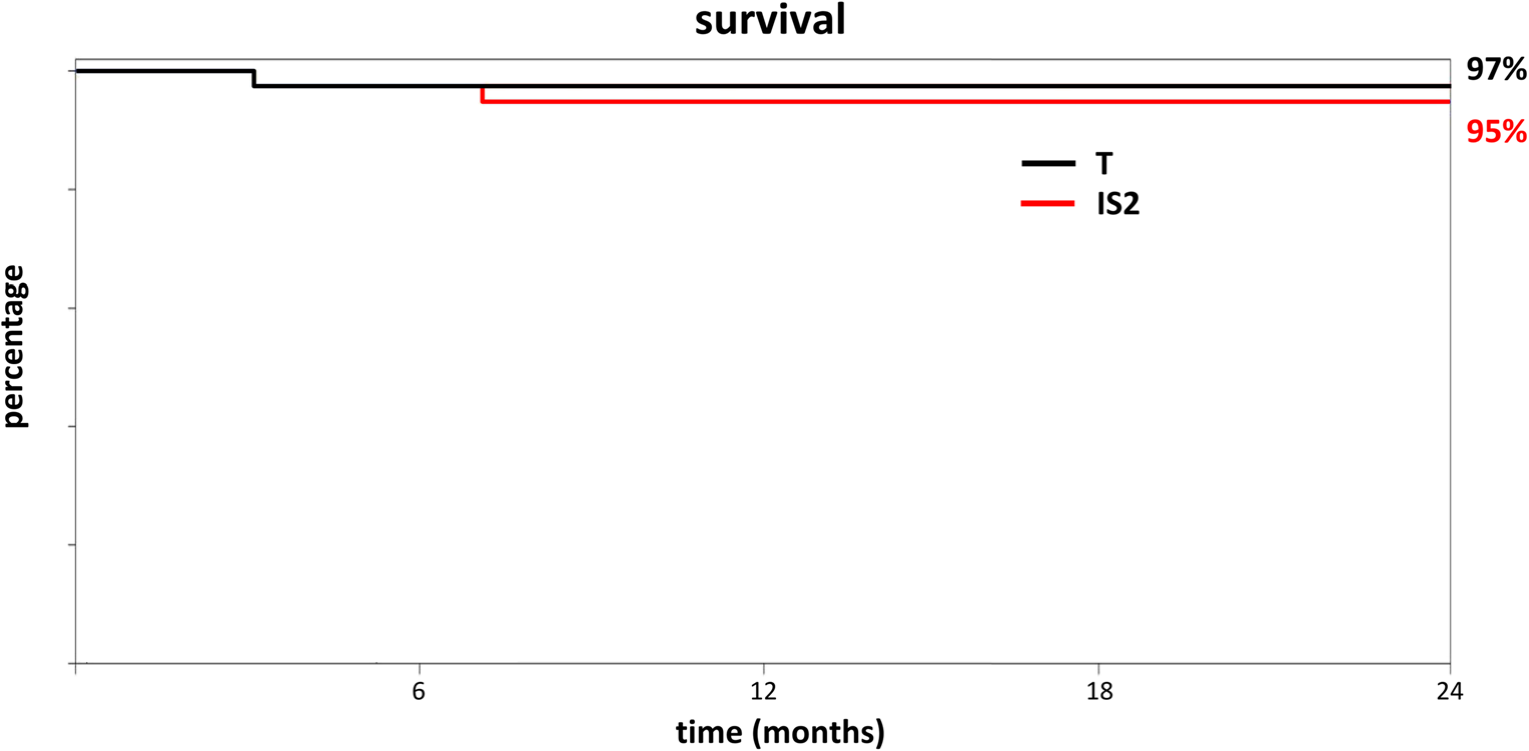
Procedure survival curve for IS2 and T with 24 months of follow-up time indicates a relatively high success rate despite a modest IOP and medication reduction. The criteria were a ≥ 20% IOP reduction *or* an IOP of less than 21 mmHg reflecting a common use pattern of IS2 as an add-on surgery at the time of cataract surgery rather than a principal glaucoma surgery

Using a definition of success commonly applied to trabecular bypass stents, a final IOP of ≤ 21 mmHg or a 20% IOP reduction from baseline, 97% of T and 95% of IS2 achieved this goal (Fig. 2). One patient in T and two in IS2 required a second surgery. No intra- or postoperative vision-threatening complications such as choroidal effusion, sustained hypotony, choroidal hemorrhage, or infection occurred.

## Discussion

This study applied exact matching to detect essential differences between IS2 and T using real-world data. Studies that use simple statistics or study design can miss those [8, 22]. Exact matching is a non-parametric method of controlling the confounding influence of pretreatment variables [23]. Each patient from one group is matched to a patient from the other group with exactly the same primary values and covariates [24, 25]. Exact matching is well-suited to compare similar pathological conditions and similar treatments, but a downside is that a certain number of datasets must be excluded from the analysis because the algorithm accepts only identical matches.

IS2 is a second-generation trabecular bypass stent [17] made of the same material as the IS1 (iStent, Glaukos Corp., San Clemente, CA) but smaller [26], allowing to fit two into one injector. The IS2 is implanted using a TM-puncturing forward movement that simplifies the technique and doubles the chance to place the device in Schlemm’s canal. In contrast, T eyes do not retain an implant as the TM is molecularized using plasma [18, 27].

In the IS2 patients followed here, we observed a slow loss of efficacy after 6 months. By 24 months, the IOP became indistinguishable from preoperative values and appeared to trend towards exceeding it. In contrast, T had a 20% reduced IOP through the study. Medications in IS2 were increased around 6 months but not above the preoperative level. In T, medications declined throughout the study. Our experience with the IS2 resembled the IS1 [3] except that IS1 patients had an IOP increase above baseline by 15% despite counteracting this trend by using 30% more glaucoma medications compared with the matched T eyes.

IS2 and IS1 are safe add-on procedures that were approved in conjunction with cataract surgery [17, 28], and this use pattern continues to be the most common one. In contrast, T is used across a spectrum of glaucoma severity [29, 30], after failed glaucoma surgery [14, 31], on its own [29], or combined with cataract surgery [30]. The survival rates we computed imply a high clinical success rate for both IS2 and T, but they are based on use criteria of an IOP below 21 mmHg or an IOP reduction by 20% commonly applied for minimally invasive glaucoma surgery (MIGS). The clinical utility of a medication reduction by 0.7 as achieved here with the IS2 depends on the motivation and goal of the affected individual. It is possible that the progressive loss of effectiveness is related to the fibrosis [1] and biofilm deposition described previously [2]. Although one might expect a more substantial effect from implanting two trabecular bypass IS2 stents than one IS1, the smaller aperture of the IS2 may make it more vulnerable to TM reactivity [9–12].

The lowest IOP any TM bypass or ablation surgery could theoretically achieve is limited by the episcleral venous pressure of about 8 mmHg present in the episcleral venous veins [32]. This limit avoids an excessive pressure reduction that might cause hypotony as can happen after traditional glaucoma surgery [33] and after suprachoroidal [34] or subconjunctival MIGS [35]. It remains unclear why trabecular bypass, disruption, or ablation methods do not routinely achieve a postoperative IOP around 8 mmHg. It is possible that post-trabecular resistor elements that have been observed in laboratory studies might play a role [36, 37].

Our study had several limitations. It was specifically designed to detect differences between IS2 and T which cannot easily be discovered by other analysis methods. Because of this, the IOP and medication changes seen here should not readily be generalized to patients who undergo these procedures but have other baseline parameters. For instance, patients with a high preoperative IOP undergoing T typically have a much more substantial IOP reduction than those with a low preoperative IOP, as examined here [29, 30]. All patients also had phacoemulsification, which can lower IOP on its own [28]. It has been speculated that the IOP reduction after phacoemulsification is caused by a trabeculoplasty-like reaction [3, 38]. IS2 might have been inadvertently helped by this additional effect [39]. In T in contrast, there is no significant contribution from cataract surgery to IOP reduction [40, 41]. Presumably, this is the case because most of the conventional outflow occurs through the 120 to 180° of nasal, unroofed Schlemm’s canal and is unaffected by the remaining trabecular meshwork [42]. However in IS2, the nasal Schlemm’s canal remains covered by glaucomatous TM and can be impacted by a trabeculoplasty-like effect of cataract surgery.

In summary, a highly balanced comparison of IS2 and T by exact matching showed that reduction of IOP and the number of glaucoma medications is lower and sustained in T. IOP in IS2 rebounds 6 to 24 months after surgery, eventually reaching preoperative levels.
